# Molecular detection of *Leptospira* spp. in rats as early spatial predictor for human disease in an endemic urban area

**DOI:** 10.1371/journal.pone.0216830

**Published:** 2019-05-22

**Authors:** Maysa Pellizzaro, Camila Marinelli Martins, Ana Carolina Yamakawa, Diogo da Cunha Ferraz, Vivien Midori Morikawa, Fernando Ferreira, Andrea Pires dos Santos, Alexander Welker Biondo, Helio Langoni

**Affiliations:** 1 Department of Veterinary Hygiene and Public Health, School of Veterinary Medicine, Sao Paulo State University, São Paulo, Brazil; 2 Department of Nursing and Public Health, Ponta Grossa State University, Paraná, Brazil; 3 Department of Preventive Veterinary Medicine and Animal Health, School of Veterinary Medicine, University of São Paulo, São Paulo, Brazil; 4 Department of Veterinary Medicine, Federal University of Paraná, Curitiba, Paraná, Brazil; 5 Zoonoses Surveillance Unit, City of Curitiba, Paraná, Brazil; 6 Department of Community Health, Federal University of Paraná, Curitiba, Paraná, Brazil; 7 Department of Comparative Pathobiology, College of Veterinary Medicine, Purdue University, West Lafayette, Indiana, United States of America; Mahidol University, THAILAND

## Abstract

**Background:**

Leptospirosis is considered a neglected zoonosis associated with infrastructure problems and low socioeconomic status, particularly slums. Since the disease is mainly transmitted in urban settings by rat urine, this risk factor may be important predictor tool for prompt control and effective prevention at the local level in urban endemic areas. Accordingly, the present study aimed to propose an early spatial predictor tool for human leptospirosis in urban settings, to test the methodology of molecular methods for assessing *Leptospira* spp. in trapped rats, and report associated environmental data.

**Methodology/Principal findings:**

Official city records and previous study were used to select risk factors for human leptospirosis in an endemic neighborhood of Curitiba, Brazil. Neighborhood census sectors were divided in high- and low-risk areas using 12 selected factors: flood area, water supply, water course, green coverage, afforestation, sewage network, open sewage, open garbage, garbage collection, dumpster, pavement, and rodent complaints. In addition, rats were captured in pre-determined sites from January through March 2017, euthanized, and individual kidneys samples sent for molecular diagnosis. Human cases were obtained from official city records. In total, 95/112 (84.8%) census sectors were classified as low-risk to human leptospirosis. No significant statistical differences were found in human case frequencies between high and low-risk areas. Kidney samples from 17/25 (68.0%) trapped rats were positive for *Leptospira* spp. The main risk factors associated with rodent presence included inadequate water supply (p = 0.04), sanitary sewage (p = 0.04), unpaved streets (p = 0.04), and complaint of rodents (p = 0.04).

**Conclusions/Significance:**

This study offers a new approach to score leptospirosis transmission risk, and to compare small areas and their heterogeneity in the same census sector of endemic areas. Environmental risk factors for *Leptospira* spp. transmission within the neighborhood were mainly due to differences in infrastructure and basic services. To the author’s knowledge, this is the first study using *Leptospira* spp. in rats as predictor for human disease in an urban setting of a major city. Although the number of rats trapped was low, this methodology may be used as basis for early and effective interventions, focused on high risk areas for leptospirosis prior to human cases, and potentially reducing morbidity and mortality in low-income areas of urban settings.

## Introduction

Leptospirosis is a reemerging zoonotic disease with approximately 350,000–500,000 severe human cases reported annually worldwide, a figure which may be underestimated due to inaccurate diagnosis and notification [1,2]. The disease has been associated with precarious infrastructure and unfavorable socioeconomic conditions, which may predispose human contact with Norway rats (*Rattus norvegicus*), reportedly the main reservoir of leptospirosis in urban settings [1,3–5].

Synanthropic rats have played an important role in spreading *Leptospira* spp. in poor urban areas, particularly in Brazilian slums [6], leading to local zoonoses units to routinely use chemical rodenticides to indirectly control leptospirosis incidence [7,8]. In addition to insufficient infrastructure and lack of basic sanitation, other risks such as proximity to open sewage, inadequate waste disposal, heavy rainfall and flooding, have reportedly favored leptospiral infections [9–11].

Microenvironment differences may substantially influence the transmission of leptospirosis due to local spatial impact of interventions such as rat and/or flood control [12,13]. Thus, small scale measures may provide a better understanding of disease dynamics and specific approaches for a given area [13]. As rats may be infected but not seroconverted at the onset of disease, molecular methods such as PCR have been used for more accurate diagnosis of *Leptospira* spp. infections in rats, by testing urine and/or kidney tissue [9,14]. Theoretically, this method of disease detection could be used as an early predictor for human exposure and disease, particularly in low-income urban areas. The aim of this study is to assess areas of high and low risk for human leptospirosis by molecular detection in rats, compared with local spatial analysis, using publicly available data and rodent information.

## Methods

### Ethics statement

Capture and use of rats in this study was approved by the Ethics Committee on Use of Animals (ECUA), protocol number 0034/2017, of the São Paulo State University (*FMVZ*, *UNESP*, *Botucatu*) which has authorized the capture, anesthesia, and euthanasia of rodents. Additionally, the study has been approved by the Curitiba City Secretary of Health. The regulation is according the National Animal Experimentation Control Council of the Brazilian Ministry of Science and Technology (Law number 11,794/2008).

### Study area

The present study was conducted in the Cajuru neighborhood (96,200 inhabitants), located in northern Curitiba (25°25'47"S, 49°16'19"W), the eighth most populous city in Brazil with approximately 1,751,907 inhabitants. The area is characterized by heterogeneous, low-income settlements with leptospirosis endemic and disease-free areas, and was chosen due to recent reports of human leptospirosis.

The census sector (CS) is defined by the Brazilian government as the territorial unit for cadastral control (used as the basis for the national census conducted every 10 years and the last one was in 2010), formed by a continuous area located in a single urban or rural space. It is considered to be the smallest official territorial unit with available information of spatial population and social data, is considered homogeneous, and is used as the sampling unit for this study. Databases were obtained from the Curitiba Institute of Research and Urban Planning (IPPUC) [15], Brazilian Institute of Geography and Statistics (IBGE) [16], and Curitiba City Hall. The average size of each CS in the Cajuru neighborhood was 104,801 m^2^ (range 19,902 to 747,955 m^2^), and the average number of households was 268 (range 21 to 655).

### Human cases

Documentation of cases of human leptospirosis was based on clinical-epidemiologic-laboratory confirmation by the Curitiba City Secretary of Health. The criteria to confirm cases was the Brazil Ministry of Health guidelines [17]. We selected the cases from the years 2014 and 2015 to test the methodology because of the completeness of the data.

### Environmental risk factors

Risk factors for leptospirosis transmission were initially selected from the available literature on environmental determinants, particularly a recent systematic review and meta-analysis which evaluated risk factors and odds ratios for each factor, according to localities on different continents [11]. Thus, relevant risk factors for local urban settings were selected from this review, using only South America data due to socio-cultural similarities [11].

Since the CS was the sample unit used, risk factors related to individual behavior and/or rural environment were removed from analysis. Thus, the twelve risk factors for the census sectors included in this study were area flooding (heavy rain caused floods), water supply (connected to public water network), water course (underground waterways), green coverage (public space with city recognition of green coverage), afforestation (city recognized tree clusters), sewage network (connected to public sewage network), open sewage (lacking canalized sewage), open garbage (any accumulated garbage), garbage collection (public waste collection), dumpster (public dumpsters present), pavement (streets were paved or not, and contact with mud) and complaints of rodents (previous rodent notification).

Risk factors were attributed to corresponding CS, thus each sector varied by the number of risk factors, from one to twelve. The CS was divided into four categories: (A) area with risk factor and with human leptospirosis cases, (B) area with no risk factor but with human leptospirosis cases, (C) area with risk factors but no human leptospirosis cases, and (D) area with no risk factors and no human leptospirosis cases. For analysis purposes, as well as to compare the frequency of positive rats and human cases, A and B were considered high-risk areas and C and D areas with low risk for human leptospirosis transmission. Thus, there were two distinct groups to analyze: high risk and low risk.

### Rodent capture and sample collection

Four CS in each of the two risk groups were randomly selected, with traps were placed in locations based on CS area, with 30 meters between each trap (distance based on the average rat travel around the colony), to ensure a representative sample of the rat population using ten to fifteen trapping locations per CS.

Tomahawk-like traps, recommended for small rodents, were initially placed and left open with bait for three consecutive days (despite rats entering) to avoid subsequent trapping failure due to neophobic behavior. Traps were inspected daily. When rats were inside, they were transported to the laboratory. If traps were disarmed, they were reset and left in place until the tenth day to provide more opportunities to catch a rat. The trapping was carried out from January through March 2017.

Once at the laboratory, rat traps were carefully placed into a plastic box and rats were anesthetized using isoflurane gas, followed by maintenance with individually adapted masks. Animals were weighed, sexed, and blood samples were obtained by intracardiac puncture. Euthanasia followed, using potassium chloride overdose, and kidney tissue was collected for analysis.

### Leptospira molecular diagnosis

A commercially available kit (Illustra, GE Healthcare, Chicago, IL, USA) was used for rat kidney DNA extractions. The polymerase chain reaction (PCR) was performed using a pair of primers that targeted the *Leptospira* spp. 16S ribosomal gene, which does not differentiate between pathogenic and saprophytic species, as previously described [18,19]. For pathogenicity confirmation, a conventional PCR was performed using a pair of primers (LipL32-45F and LipL32-286R) [20], that targeted the *Leptospira* spp. LipL32 pathogenic gene.

### Statistics and geographic data

As rodents are potential *Leptospira* spp. carriers, frequencies of positive rats were exclusively based on kidney PCR results. Data was analyzed using descriptive statistics and association with sex and age verified by chi-square or Fisher’s exact test. Results were considered statistically significant when p-values were ≤ 0.05. Tests of association with each risk factor was individually performed and the intensity of association evaluated by odds ratio and 95% confidence interval using the SPSS software (2008).

Geographic coordinates were registered in each place of rodent capture using a commercial global positioning system (GPSMAP 64S, Garmin Ltd, Olathe, KS, USA). Outcome data were plotted on maps to compare the spatial distribution of human cases in a commercial GIS software (ArcGIS 10.1, Esri, Redlands, CA, USA).

## Results

**Fig 1:**
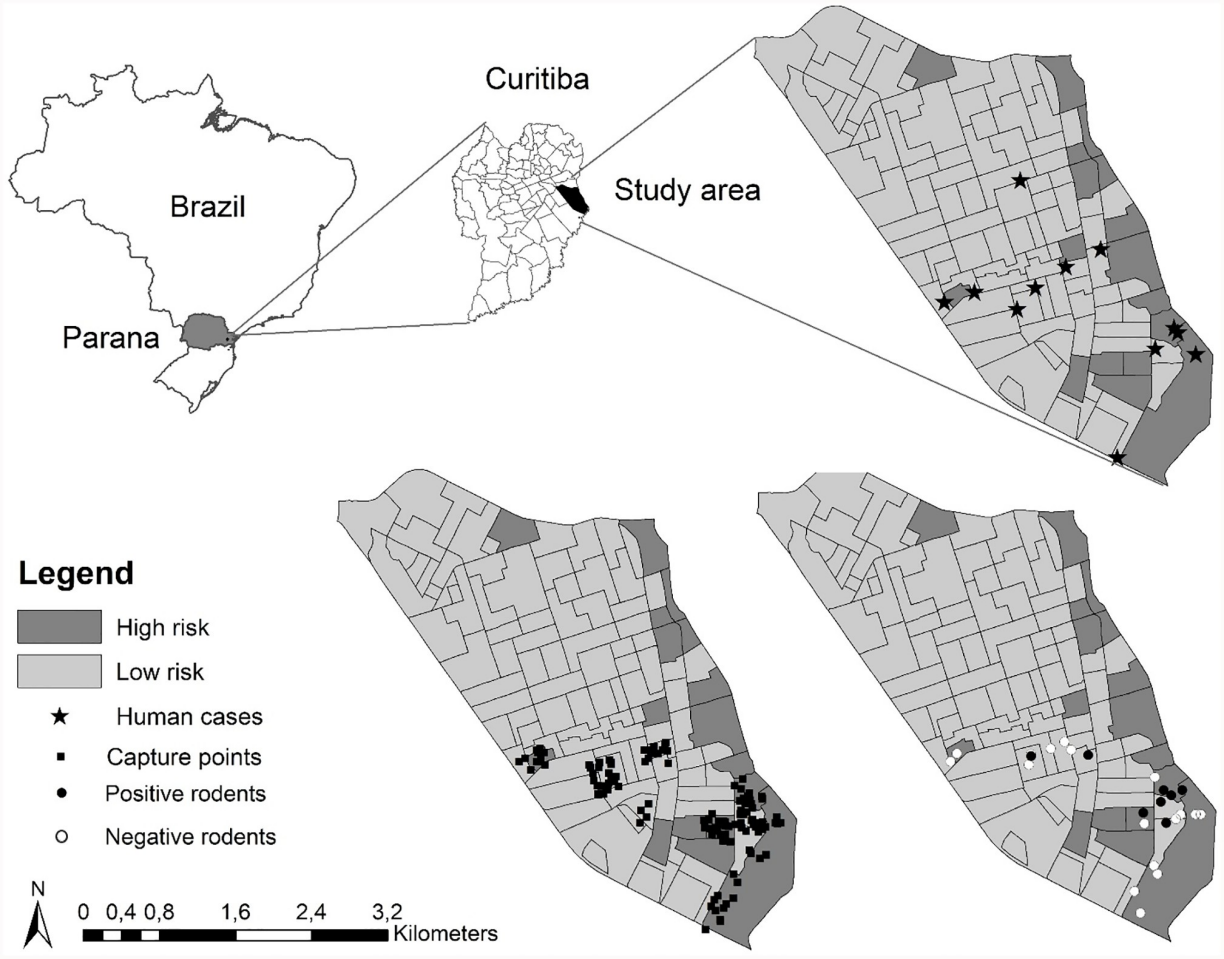
Localization of the city of Curitiba, Brazil and the Cajuru neighborhood within the city. Neighborhood maps show locations of the 12 human leptospirosis cases in 2014 and 2015 in high- and low-risk areas (top), the 129 trapping locations randomly selected for two census sectors of each group (bottom left), and the locations of 25 trapped rats (bottom right). Maps were divided into high-risk (dark gray) and low-risk (light gray) areas for human leptospirosis transmission. The maps were produced by authors, using free open access shapefiles described in methodology section: IPPUC [15] and IBGE [16] and performed on GIS software.

All of the 112 CS of the Cajuru neighborhood were considered and classified by number of risk factors, and ranged from zero to seven (Fig 1). The CS with up to three risk factors were considered to be low risk, and those with four to seven risk factors were considered to be high risk, for human leptospirosis transmission. The sum of risk factors for the 112 sectors and the classification into high and low risk areas is presented (Fig 1). Overall, 17/112 (15.2%) of CS were classified as high risk, and 95/112 (84.8%) of CS were classified as low risk of disease transmission, with a maximum of 7/12 (58.3%) risk factors simultaneously found in the same census sector.

A total of 42/112 (37.5%) CS were located in flooding areas, only 3/112 (2.6%) had houses without water supply, and 43/112 (38.3%) were located in water course area. In addition, 26/112 (23.2%) had green coverage, 101/112 (90.1%) were considered forested, 109/112 (97.3%) had all houses connected to public sewage, 44/112 (39.2%) had open sewage present, 38/112 (33.9%) had some open garbage, 112/112 (100%) had public garbage collection, 30/112 (26.7%) had local dumpsters, 109/112 (97.3%) CS had paved streets, and 81/112 (72.3%) had present rodent complaints.

The distribution of human leptospirosis cases in 2014 and 2015 in CS were assessed and presented (Fig 1), with 2/17 (11.8%) high risk areas and 9/95 (9.5%) low risk areas having leptospirosis cases. Although the proportion of cases was higher among high-risk sectors, no statistical difference was found (p = 0.08). The absence or presence of each risk factor in high- and low-risk areas and respective frequencies were also presented (Table 1).

**Table 1:** Presence or absence of risk factors on census sectors and occurrence of human leptospirosis cases in 2014 and 2015, in Cajuru neighborhood (Curitiba, Paraná, Brazil).

	Human leptospirosis cases			
Risk factors		Frequency (%)	p-value	OR (CI 95%)
Flood area	Yes	6/42 (14.3)	0.21	2.16 (0.61–7.59)
	No	5/70 (7.1)		
Water supply	Yes	10/109 (9.2)	0.16	4.95 (0.41–59.52)
	No	1/3 (33.3)		
Water course	Yes	6/43 (14.0)	0.24	2.07 (0.59–7.27)
	No	5/69 (7.2)		
Green coverage	Yes	2/26 (7.7)	0.67	0.71 (0.14–3.52)
	No	9/86 (10.5)		
Afforestation	Yes	8/101 (7.9)	0.04	0.22 (0.05–1.03)
	No	3/11 (2.3)		
Sewage network	Yes	10/109 (9.2)	0.16	4.95 (0.41–59.52)
	No	1/3 (33.3)		
Open sewage	Yes	5/44 (11.4)	0.65	1.32 (0.37–4.63)
	No	6/68 (8.8)		
Open garbage	Yes	3/38 (7.9)	0.62	0.70 (0.17–2.83)
	No	8/74 (10.8)		
Garbage collection	Yes	11/112 (9.8)	-	-
	No	-		
Dumpster	Yes	2/30 (6.7)	0.49	1.72 (0.35–8.48)
	No	9/82 (11.0)		
Pavement	Yes	10/109 (9.2)	0.16	4.95 (0.41–59.52)
	No	1/3 (33.3)		
Rodent complaint	Yes	8/81 (9.9)	0.97	1.02 (0.25–4.13)
	No	3/31 (9.7)		

Rodent traps were placed in a total of 129 different locations from 8/112 (7.14%) randomly selected CS. Rats were captured in 25/129 (19.3%) traps over an average of 3.16 (range 1–10) nights. The traps were placed to ensure coverage of all of the CS area, and to avoid capturing more than one rat from each colony in order to obtain a representative sample (S1 Dataset). A total of 25 rats were trapped, 17 females and 8 males, and all were *Rattus norvegicus* species. A total of 17/25 (68.0%) rats were positive by PCR amplification of *Leptospira* spp. using the universal primer, and 14/25 (56.0%) were positive using a LipL32 gene of *Leptospira* spp. The fourteen positive rats using the specific pathogenic primer were obtained with the same the universal protocol. For analysis purpose, we use the results from the universal primer due to the low chronic infection of saprophytic Leptospira in rat’s kidneys. Although females presented higher frequency than males having 13/17 (76.5%) and 4/8 (50.0%) positive rats, respectively, no statistical difference was observed (p = 0.18). The frequency was higher in older animals than juvenile, with 3/7 (42.9%) and 14/18 (77.8%) of positive rats, respectively, with no statistically significant difference (p = 0.09). Frequency of positive rodents in high-risk and low risk areas and stratified analysis per risk factor were obtained and presented (Table 2).

**Table 2:** Presence or absence of risk factors on census sectors and PCR detection of *Leptospira* spp. in kidneys from trapped rats in the Cajuru neighborhood (Curitiba, Paraná, Brazil).

	*Leptospira* spp. in rat’s kidneys			
Risk factors		Frequency (%)	p-value	OR (CI 95%)
Flood area	Yes	15/20 (75.0)	0.13	4.50 (0.57–35.15)
	No	2/5 (40.0)		
Water supply	Yes	16/21 (76.2)	0.04	0.10 (0.00–1.23)
	No	1/4 (25.0)		
Water course	Yes	10/15 (66.7)	0.86	0.85 (0.15–4.81)
	No	7/10 (70.0)		
Green coverage	Yes	8/11 (72.7)	0.65	1.48 (0.26–8.26)
	No	9/14 (64.3)		
Afforestation	Yes	7/10 (70.0)	0.86	1.16 (0.20–6.55)
	No	10/15 (66.7)		
Sewage network	Yes	16/21 (76.2)	0.04	0.10 (0.00–1.23)
	No	1/4 (25.0)		
Open sewage	Yes	4/6 (66.7)	0.93	2.91 (0.28–30.29)
	No	13/19 (68.4)		
Open garbage	Yes	5/6 (83.3)	0.35	0.70 (0.17–2.83)
	No	12/19 (63.2)		
Garbage collection	Yes	17/25 (68.0)	-	-
	No	-		
Dumpster	Yes	-	-	-
	No	17/25 (68.0)		
Pavement	Yes	16/21 (76.2)	0.04	0.10 (0.00–1.23)
	No	1/4 (25.0)		
Rodent complaint	Yes	16/21 (76.2)	0.04	9.60 (0.80–114.17)
	No	1/4 (25.0)		

## Discussion

This study aimed to provide a comprehensive approach based on reliable bibliography information, official data of spatial populations, and epidemiologic information, to assess the impact of neighborhood level factors on the transmission of leptospirosis from rats to humans. This methodology may be adapted for use in different localities worldwide by both researchers and governments.

The categorization of Cajuru neighborhood into areas of high and low risk, based on census sector (CS) characteristics and absence/presence of environmental risk factors, provided a method for ranking risk, since some factors may increase the risk of disease transmission [21]. This has been reported as useful for specific interventions and for increasing awareness of populations at higher risk [22].

The present study has proposed the stratification into census sectors as worthwhile tool on basic territorial unit, successfully used for detailed socioeconomic indicators provided by the IBGE. Despite high-risk CS have presented higher frequencies herein, limitations due to small populations and low case numbers, even gathering cases through years, may have impacted on absence of significantly differences [21,23].

The few CS lacking full coverage of basic sanitary services, such as water supply and connection to public sewage network, may have exposed those populations to human leptospirosis, since open sewage, flooding areas, and accumulated garbage have been reported as risk factors of disease [4,10,24]. Not surprisingly, these same factors have been indicators to determine the human leptospirosis incidence at the local level [4,12,21].

The high number of CS with accumulated garbage (33.9%) was, probably, due to recycling material collection for family income, separated on their own properties or community sheds, mostly in an inadequate manner, increasing the chance of attracting rodents. Although several studies have suggested the association of garbage with an increase of leptospirosis cases [4,10,11,25], garbage on neighboring property was not a risk factor in a previous study performed in Salvador, northeastern Brazil [24].

The lack of pavement was used as an indicator of mud contact following rain, previously described as a risk factor for leptospirosis transmission in an endemic area [13,24]. The majority of CS had all streets pavement (97.3%), but several streets had no pavement in some CS, mainly near the river, where flooding drainage systems to absorb large volumes of rain may expose affected population, a well-known risk factor [24,25].

Synanthropic rodents have been considered the main reservoirs of *Leptospira* spp. which may be viably shed in rat urine in both risk and no-risk areas. Thus, risk factors have played a role in predisposing susceptible persons to contact with contaminated urine [13,24,25]. Records of rodent complaints received by the Zoonosis Surveillance Unit were used as proxy for local presence of rats in our study [25,26]. As expected, the majority of CS had recorded rodent complaints (72.3%). A previous study has shown that observation of five or more rats within the neighborhood was a risk factor for leptospirosis transmission [24], while seeing two or more rats was implicated with increase on serum prevalence [4]. As already established, control of rodents in high-risk areas should be an important measure for reducing human leptospirosis incidence [4,6].

Although high-risk areas have had greater frequencies of human leptospirosis cases, no significant differences have been found between CS areas of high- and low-risk. Similar results have also been found for *Leptospira* spp. detection in trapped rats. Nonetheless, molecularly positive rats should be always considered an important finding as rodent presence itself may not indicate risk of disease or environmental dissemination of bacteria [25]. Though positive rats were mostly trapped in high-risk areas, no significant differences were found for molecular detection of *Leptospira* spp. in rats due to outdoor food and trash availability, and further studies should be performed to compare rat populations and positivity of trapped rats. Finally, though no association was found between risk factors and human leptospirosis cases, a longer study in the future may provide more rat samples and human clinical cases, increasing statistical power for comparing potential differences. We hypothesized that there would be positive animals in each of the four risk categories and what would identify potential human leptospirosis risk factors. However, contrary to one of our hypotheses, there was no association between risk factors and cases.

The molecular frequency of *Leptospira* spp. detected in rats was similar to previous studies in Salvador, northern Brazil, with frequencies greater than 80% in rats captured in a leptospirosis endemic area [27,28]. Since rats may be asymptomatic reservoirs while shedding bacteria through urine, PCR detection of *Leptospira* spp. in rat kidneys should be used for rat diagnosis. Unfortunately, studies to date have mostly assessed rats by serological diagnosis, reportedly less sensitive than molecular [1]. Regarding the difference in PCR results using distinct primers, previous studies did not perform both approaches. Still, a prior study conducted in same city, detected nonpathogenic *Leptospira* strains using serologic assays. This indicates the rat’s susceptibility to *Leptospira* infection whether they are pathogenic or not, however, further studies should use methods able to differentiate *Leptospira* spp. infecting rats’ kidneys [29].

Despite females having a higher frequency of positive samples than males, no statistical difference (p = 0.18) was observed, which is probably due to similar exposure to a contaminated environment. Such absence of statistical differences on sex has been previously described [6,27,30], with a single study reporting a greater positive frequency in females [31].

Even though older rats were more often positive for *Leptospira* spp. detections than younger ones, no statistical difference (p = 0.09) was found. Similarly, higher frequencies have been previously described in adult rats, also without statistical significance, which is probably due to older rats have longer exposure in a contaminated environment, and intimate contact from social living with others rats [6,27,29, 30,32].

Test positive rodents were founded in both, high- and low-risk areas, however, sectors without water supply, sewage network connection, lacking pavement, and with previous rodent complaints have shown association with an increase in test positive rodents (p = 0.04 for each factor). Nevertheless, a higher proportion of positive rodents was not found in CS with these risk factors, which have been previously associated with increase in human cases [11,25,28]. Such similar results may have been caused by residential location on streets without pavement along absence of sewer and water networks, as such services are usually installed together.

Other limitations included the two-year period of human leptospirosis cases used for analysis, the reduced number of trapped rats, and low number of CS selected. Also, each risk factor was graded for the entire CS, despite heterogeneity within the sector, or variation in the number of households at risk of each factor. Although CS were the smallest available territorial unit, they were still large, and had variable sizes and heterogeneous populations. In general, the classification of the risk areas had similar limitations of an ecological study, since individual characteristics were also taken in account. We focused on environmental risk factors, but leptospirosis is a multifactorial disease, and further studies may also include individual’s behavior and other animals as potential reservoirs.

In conclusion, we have proposed a model to evaluate different environmental risk factors for human leptospirosis, using census sectors as an already established system to access rodents and human cases, and comparing areas of high and low risk in a major Brazilian city. To the authors’ knowledge, this is the first published approach for comparison of heterogeneous areas nearby, particularly within in an urban setting of a major city. Our findings may potentially provide better knowledge to create and/or execute intervention programs in high-risk areas, to raise quality’s life of slums population.

## Supporting information

S1 Dataset Data source, risk factors, coordinates of rodent capture and human cases and results of kidneys PCR. (XLSX)
